# Establishment of an in vitro co-infection model of *Cryptosporidium parvum* and *Giardia duodenalis*

**DOI:** 10.1186/s13071-025-06926-5

**Published:** 2025-07-12

**Authors:** Manuela Kirchner, Arwid Daugschies, Cora Delling

**Affiliations:** Institute of Parasitology, Faculty of Veterinary Medicine, Leipzig University, Leipzig, Sachsen, Germany

**Keywords:** *Cryptosporidium*, *Giardia*, Cell culture techniques, Co-infection

## Abstract

**Background:**

The two intestinal protozoan parasites *Giardia duodenalis* and *Cryptosporidium parvum* cause infections in a wide spectrum of vertebrates and have also been shown to infect suitable hosts simultaneously. To investigate potential effects between these parasites and on host cells, a co-infection model with IPEC-J2 cells was established.

**Methods:**

Optimal infection conditions and several infection doses of both parasites were tested. The effect of *Giardia* growth medium on IPEC-J2 cells was analyzed using 3-(4,5-dimethylthiazol-2-yl)-2,5-diphenyltetrazolium bromide (MTT) reduction assay, while the effect of different infection doses of each parasite on host cell viability was investigated by CellTiter Blue cell viability assay. For co-infection, IPEC-J2 cells were first infected with *C. parvum* sporozoites, and 3.5 h later, *G. duodenalis* trophozoites were added. Parasite propagation during single infection and co-infection were analyzed by quantitative real-time polymerase chain reaction (qPCR) as well as immunofluorescent staining.

**Results:**

The infection with *C. parvum* sporozoites had no significant impact on cell viability, while *G. duodenalis* trophozoites affected cell culture in a dose dependent manner. The amount of gene copies of *C. parvum* in single and co-infected cells did not differ significantly, while statistically higher amounts of *G. duodenalis* gene copies in co-infected cell cultures were identified.

**Conclusions:**

In this study, single infections and co-infections of IPEC-J2 cells with *C. parvum* and *G. duodenalis* were established and optimized over a period of 72 h.

**Graphical Abstract:**

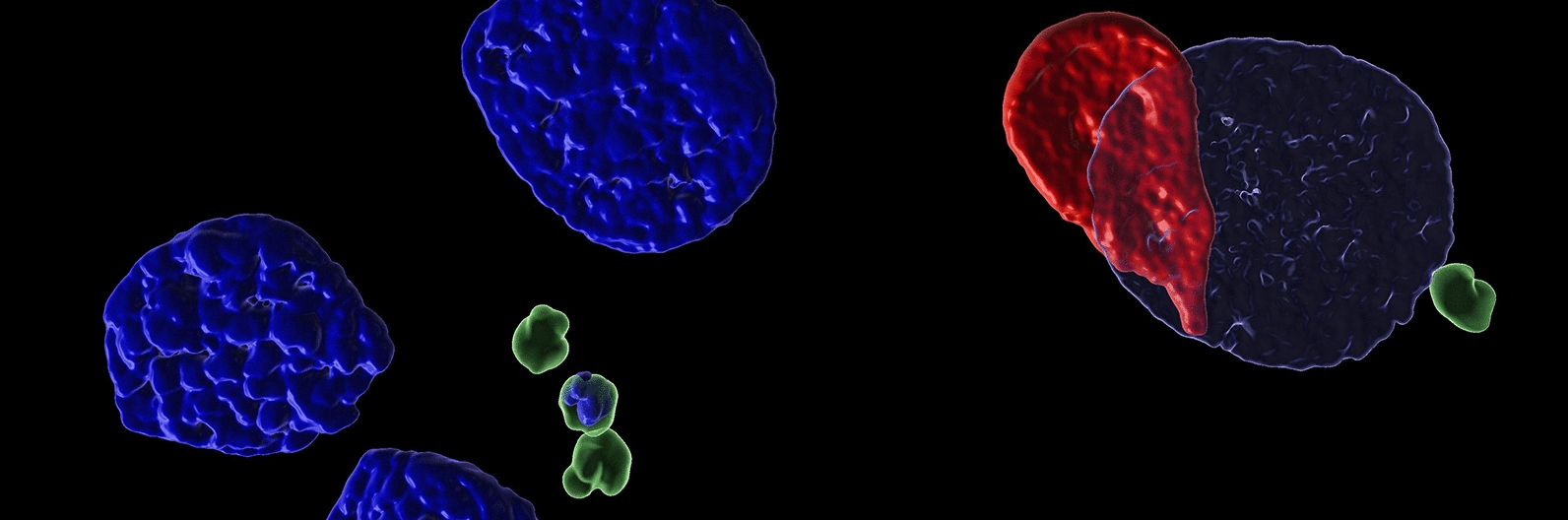

## Background

*Giardia duodenalis* (syn. *Giardia lamblia*, *Giardia intestinalis*) and *Cryptosporidium parvum* are both protozoan parasites that are able to infect a wide spectrum of vertebrates, including humans [1, 2]. *Cryptosporidium parvum* is one of the most common *Cryptosporidium* species responsible for human infections, and the dominant *C. parvum* subtypes in humans in North America, Europe, and Australia are also common bovine subtypes [3], highlighting the zoonotic potential of this pathogen. *Giardia duodenalis* can be sorted into eight groups called assemblage A–H on the basis of different genotypes [4]. After oral uptake by a suitable host, *Giardia* attaches mainly extracellularly on duodenal epithelial cells and multiplies by longitudinal binary fission [5], while *C. parvum* multiplies in epithelial cells of the small intestine intracellularly but extracytoplasmatically and undergoes different consecutive development stages divided in an asexual and a sexual reproduction cycle [6]. The cysts of *Giardia* spp. and oocysts of *Cryptosporidium* spp. are immediately infective when passed with feces and may persist in the environment for months [7], so an infection can be transmitted either by direct fecal–oral contact or indirectly by an uptake of contaminated water, food, or soil [8]. Simultaneous infections with both parasites occur in several animal species [9, 10], but the clinical relevance of these events remains unknown [11].

Venter et al. stated that the conduction of well-controlled laboratory studies with standardized circumstances as well as the investigation of co-factors is of great importance regarding the examination of virulence and transmission of pathogens [12]. Axenic culture of *Giardia* trophozoites is possible [13, 14] and is used for, e.g., disinfection testing. Co-cultures of intestinal cell lines with *Giardia* have been established before to examine interactions between host cells and parasites [15]. Furthermore, various studies demonstrated *C. parvum* infection in different cell lines [16–18] and three-dimensional (3-D) models such as organoids or stem-cell derived culture systems have been reported for this parasite [19, 20]. As a nontransformed epithelial cell line derived from neonatal piglet mid-jejunum tissue, IPEC-J2 cells exhibit similar behavior to intestinal epithelial cells [21]. Therefore, pathomechanisms of the infection with several protozoan parasites including *C. parvum* were previously investigated using this particular cell line [22–25]. To investigate the interaction between pathogens in vitro, co-infection models have been established [26], and some of these models appear suited to examine the interplay between protozoan parasites. Studies by Taha et al. and Zhang et al. showed that simultaneous infection by two species of intracellular parasites can hinder each other’s development significantly [18, 27, 28]. To examine potential interactions between the extracellular parasite *Giardia* and the intracellular parasite *Cryptosporidium* as well as possible effects of this co-infection on the host, a co-infection model was established using IPEC-J2 cells as host cells.

## Methods

### Axenic culture of *Giardia* trophozoites

Trophozoites of *G. duodenalis* WB6 (assemblage AI) were obtained from the Robert Koch Institute (Berlin, Germany) and cultured as described elsewhere [14] with some modifications. The parasites were grown under anaerobic conditions in three milliliter (ml) cell culture tubes (Nunc™, ThermoFisher Scientific, Waltham, USA) with modified TYI-S-33 medium containing 1.8 g casein peptone (Sigma-Aldrich^®^, Steinheim, Germany), 1.0 g d(+)-glucose (Carl Roth GmbH, Karlsruhe, Germany), 0.9 g yeast extract (Sigma-Aldrich^®^, Steinheim, Germany), 200 mg sodium chloride (Carl Roth GmbH, Karlsruhe, Germany), 100 mg K_2_HPO_4_ (Carl Roth GmbH, Karlsruhe, Germany), 60 mg KH_2_PO_4_ (Carl Roth GmbH, Karlsruhe, Germany), 20 mg l(+)-ascorbic acid (Carl Roth GmbH, Karlsruhe, Germany), 10 ml heat-inactivated fetal calf serum (PAN Biotech™, Aidenbach, Germany), 200 mg l-cysteine (Sigma-Aldrich^®^, Steinheim, Germany), 52 mg dried, unfractionated bovine bile (Sigma-Aldrich^®^, Steinheim, Germany), and 2.28 mg ammonium-iron(III) citrate (Carl Roth GmbH, Karlsruhe, Germany) in 100 ml. The medium was sterile filtered before use with Filtropur S 0.2 (Sarstedt AG&Co. KG, Nümbrecht, Germany).

To initiate subcultures, the medium containing unattached and dead parasites in tubes with confluent *Giardia* cultures was removed. Afterwards, the tubes were refilled with 6 ml freshly prepared medium and incubated on ice for 15 min . After the following centrifugation step at 650 × *g* for 10 min at 4 °C, the pellet was resuspended in 1 ml medium and motile trophozoites were counted in a Neubauer chamber at 200× magnification. Subcultures were initiated by inoculating a new culture tube containing 10.5 ml freshly prepared medium with 10^4^–10^5^ trophozoites.

### Maintenance of *Cryptosporidium parvum* oocysts

For all experiments, an in-house isolate of *C. parvum* (LE-23-Cp-23/1, Markranstädt, containing the *gp60* subtypes IIaA15G2R1, IIaA12G2, IIaA14G2R1) was used. The maintenance of the parasites was assured through passages in neonatal calves every 3–6 months. The oocysts were isolated and stored as described earlier [29, 30]

### Maintenance of IPEC-J2 cells

IPEC-J2 cells (passage 45–56) were kindly provided by the Institute of Physiology, University of Veterinary Medicine Hannover and were used for all experiments. Cultivation of cells was performed as described elsewhere [24] with some modifications. For infection, 2 × 10^5^ cells were seeded in each well of 24-well plates, covered with growth medium (GM) consisting of Iscove’s modified Dulbecco’s medium (IMDM; Gibco™, ThermoFisher Scientific, Waltham, USA), supplemented with 50% Ham’s *F*-12 nutrient mixture W/GlutaMAX™-I (*F*-12; Gibco™, ThermoFisher Scientific, Waltham, USA), 10% fetal calf serum (FCS; PAN Biotech™, Aidenbach, Germany), 0.002 mM l-glutamine (Gibco™, ThermoFisher Scientific, Waltham, USA), 0.5 U/ml penicillin, 0.5 µg/ml streptomycin (Gibco™, ThermoFisher Scientific, Waltham, USA), and 0.025 ml amphotericin B (PAN Biotech™, Aidenbach, Germany) per ml and grown at 37 °C, 5% CO_2_ for 72 h.

### MTT assay

To test the influence of *Giardia* medium on the viability of IPEC-J2 cells, a MTT assay (Carl Roth GmbH, Karlsruhe, Germany) was performed as previously described [31] with some modifications. The cells were seeded into 96-well cell culture microplates (1 × 10^4^ cells/well) and grown for 24 h (37 °C, 5% CO_2_) to produce confluent monolayers (confluence > 90%). Afterwards, the cell cultures were washed once with phosphate-buffered saline (PBS; Gibco™, ThermoFisher Scientific, Waltham, USA), and 100 µl of GM supplemented with either 10%, 30%, or 50% of TYI-S-33 medium or TYI-S-33 medium only was added to confluent monolayers. Additionally, three wells containing PBS served as a positive control (PC), while cells in three wells were grown in GM (NC). The viability of the cells was tested 24 h, 48 h, and 72 h after GM or supplemented GM was applied. For this purpose, GM was replaced by IMDM, supplemented with 50% *F*-12 without any other additives. MTT solution (10 µl, 0.5% thiazolyl blue, Carl Roth GmbH, Karlsruhe, Germany) was added, and incubation was continued for another 4 h. Afterwards, 100 µl of 10% sodium dodecyl sulphate (Carl Roth GmbH, Karlsruhe, Germany) in 10 mM hydrochloric acid was added to each well to stop further processing of MTT. The microplates were then incubated at 37 °C and 5% CO_2_ overnight before optical density (OD) values were analyzed at 595 nm for each well using a BioTek 800 TS microplate reader (BioTek Instruments GmbH, Bad Friedrichshall, Germany). Every assay was performed in triplicates. Three cell-free wells contained medium only and served as blank control (BC). Cell viability of cultures was calculated based on OD as follows:

$${\text{cell viability}} \left( \% \right) = \frac{{{\text{OD isolate}} - {\text{ OD BC}}}}{{{\text{OD NC }} - {\text{OD BC}}}},$$

where OD NC is the mean OD value of triplicate NC and OD BC is the mean OD value of triplicate BC. To ensure reproducibility, the experiments were repeated four times.

### Infection of IPEC-J2 cells with *C. parvum*

Excystation of oocysts was performed as described before [32]. Briefly, oocysts were bleached with 4 °C cold 2.625% sodium hypochlorite (NaOCl) in PBS (Gibco™, ThermoFisher Scientific, Waltham, USA) for 5 min on ice and washed three times with PBS afterwards. The next steps included the resuspension of decontaminated oocysts in excystation medium consisting of infection medium (see below) and 0.4% taurocholic acid (Sigma, Steinheim, Germany) and incubation at 15 °C for 1 h. Thereafter, the oocysts were incubated for another hour at 37 °C and 5% CO_2_. Excysted sporozoites were counted and suspended in infection medium consisting of Dulbecco’s modified Eagle’s medium (DMEM; Gibco™, ThermoFisher Scientific, Waltham, USA) supplemented with 2% FCS, 0.5 U/ml penicillin, 0.5 µg/ml streptomycin (Gibco™, ThermoFisher Scientific, Waltham, USA), 0.025 ml amphotericin B (PAN Biotech™, Aidenbach, Germany), 0.01 mM sodium pyruvate (ThermoFisher Scientific, Waltham, USA), and 0.04 mM l-glutamine (Gibco™, ThermoFisher Scientific, Waltham, USA) per ml. The IPEC-J2 cells were washed three times with 37 °C preheated PBS, and thereafter, 1 ml infection medium containing either 2 × 10^5^, 4 × 10^5^ or 8 × 10^5^ sporozoites was added to each well. The infected cultures were immediately centrifuged at 800 × *g* for 5 min at room temperature (RT) and then incubated for 3.5 h (37 °C, 5% CO_2_), before the infection medium was removed and 1 ml of GM was added. The cells were harvested either 24 h post infection (p.i.), 48 h p.i. or 72 h p.i. To ensure reproducibility, experiments were performed six times. Monolayer integrity was visually assessed by light microscopy (200× magnification) at all timepoints mentioned above.

### CellTiter Blue cell viability assay following *C. parvum* mono-infection

To test the influence of different infection doses on the viability of IPEC-J2 cells, a CellTiter Blue cell viability assay (Promega, Mannheim, Germany) was conducted. The cells were seeded in a 24-well plate and infected with *C. parvum* sporozoites as described above. Noninfected cells grown in GM were used as NC, and noninfected cells treated with PBS served as PC. At 24 h p.i., 48 h p.i., or 72 h p.i., the medium was removed and cells were washed three times with PBS first, before 400 µl of IMDM substituted with 50% *F*-12 was added. Then, 80 µl of CellTiter-Blue^®^ reagent (Promega, Mannheim, Germany) was added to each well. After 2 h of incubation (37 °C, 5% CO_2_), 100 µl of medium was collected from each well and absorbance at 570 nm as well as 630 nm was measured by using a Synergy H1 microplate reader (BioTek Instruments GmbH, Bad Friedrichshall, Germany). For calculating the cytotoxicity of different infection doses, the values obtained were normalized to uninfected control (NC) cells according to the equation:

$$\frac{{\left( {O2 \times A1} \right) - (O1 \times A2)}}{{\left( {O2 \times P1} \right) - (O1 \times P2)}} \times 100.$$

(taken from https://www.bio-rad-antibodies.com), where O1 is the molar extinction coefficient of oxidized alamarBlue^®^ at 570 nm, O2 is the molar extinction coefficient of oxidized alamarBlue^®^ at 630 nm, A1 is the absorbance of test wells at 570 nm, A2 is the absorbance of test wells at 630 nm, P1 is the absorbance of growth control well (noninfected cells with CellTiter-Blue^®^) at 570 nm, and P2 is the absorbance of growth control well (noninfected cells with CellTiter-Blue^®^) at 630 nm.

### Infection of IPEC-J2 cells with *G. duodenalis* trophozoites

IPEC-J2 cells were prepared as described above and washed three times with PBS before infection. Axenically grown *G. duodenalis* trophozoites were harvested and counted as described above. Trophozoites were then transferred to IPEC-J2 cells covered with GM containing 10% TYI-S-33. Four different infection doses were used (1 × 10^4^, 5 × 10^4^, 1 × 10^5^, and 2 × 10^5^ trophozoites/well), and the infected cell cultures were washed three times with PBS to remove nonadherent trophozoite directly before being sampled 24 h p.i., 48 h p.i., and 72 h p.i. To ensure reproducibility, experiments were performed six times.

Monolayer integrity and attachment of *G. duodenalis* trophozoites was visually assessed by light microscopy (200× magnification) at all timepoints.

### CellTiter Blue cell viability assay of *G. duodenalis* mono-infection

To test the influence of several infection doses over 72 h p.i. on the viability of IPEC-J2 cells, a CellTiter Blue cell viability assay (Promega, Mannheim, Germany) was conducted. The cells were seeded in a 24-well plate and infected with *G. duodenalis* trophozoites as described above. Noninfected cells grown in GM were used as NC, and noninfected cells treated with PBS served as PC. The medium was removed 72 h p.i., and cells were washed three times with PBS first, before IMDM substituted with 50% *F*-12 and 40 µM Formononetin (Sigma-Aldrich^®^, Steinheim, Germany) was added to each well. After 30 min of incubation (37 °C, 5% CO_2_), cells were washed three times with PBS to remove all nonadherent trophozoites, and the assay was continued as described above.

To ensure no trophozoites were left to falsify the viability assay results, cells were thoroughly assessed by light microscopy (200× magnification) before the assay was conducted.

### Co-culture model

For co-infection, IPEC-J2 cells were seeded in 24-well plates and grown to confluence. Monolayers were infected with *C. parvum* sporozoites 72 h later as described above. After 3.5 h of infection with *C. parvum*, *G. duodenalis* trophozoites suspended in GM with 10% TYI-S-33 were applied to the cell cultures. After 24 h p.i., 48 h p.i., and 72 h p.i. with *G. duodenalis*, the co-infected cultures were harvested. To ensure reproducibility, experiments were performed six times.

Monolayer integrity and attachment of *G. duodenalis* trophozoites was visually assessed by light microscopy (200× magnification) at all timepoints.

### DNA extraction and quantitative real-time PCR (qPCR)

Cells washed three times with PBS were harvested by trypsin digestion (1×) (PAN Biotech™, Aidenbach, Germany) for 15 min (37 °C, 5% CO_2_). Two wells were subsequently pooled, resuspended in 200 µl PBS, and stored at −20 °C until DNA extraction. DNA extraction was performed with QIAmp DNA Mini Kit (QUIAGEN, Hilden, Germany) according to the manufacturer’s protocol. Total DNA was photometrically quantified with NanoPhotometer^®^ NP80 (Implen, Munich, Germany) and adjusted to 70 ng/µl.

*C. parvum* infection was quantified by qPCR addressing the Cp hsp70 gene as described in Refs. [30, 33]. Briefly, for real-time PCR assay, 12.5 µl of Rox qPCR Master Mix (×2) (ThermoFisher Scientific, Waltham, MA, USA), 0.3 µM of the forward primer (CP_hsp70_fwd (2219–2246) 5′-aactttagctccagttgagaaagtactc-3′), 0.9 µM of the reverse primer (CP_hsp70_rvs (2336–2362) 5′-catggctctttaccgttaaagaattcc-3′), 0.2 µM of the TaqMan probe (HSP70 5′-FAM aatacgtgtagaaccaccaaccaatacaacatc BHQ1-3′), and 5 µl of template filled with DNA/nuclease-free water up to a volume of 25 µl was used.

*G. duodenalis*-infection was quantified by qPCR addressing the β-giardin gene as described by Bertrand et al. [34]. Briefly, a 95-bp fragment was amplified with 12.5 µl Rox qPCR Master Mix (×2) (ThermoFisher Scientific, Waltham, MA, USA), 0.5 µM of both primers, the forward primer (G-for (776–798) 5′-TCTATGTTCACCTCCACCCGTAC-3′) and reverse primer (G-rev (853–870) 5′-TTGCTGAGCTT GACCGCC-3′), 0.3 µM of the TaqMan probe (G-probe (801–823) 5′-HEX TCACCCAGACGATGGA CAAGCCC BHQ1-3′), and 5 µl of template filled with DNA/nuclease-free water up to a volume of 25 µl.

All primers were obtained from Invitrogen (ThermoFisher Scientific, Waltham, MA, USA), and all experiments were conducted using a Bio-Rad CFX Connect real-time PCR detection system (Bio-Rad, Feldkirchen, Germany).

### Immunofluorescence assay

IPEC-J2 monolayers were exposed to both parasites in 24-well plates on glass cover slips under the same conditions as described above. At 24 h p.i., 48 h p.i., and 72 h p.i. the cells were washed three times with Dublecco’s phosphate-buffered saline w: Ca/Mg (DPBS; PAN Biotech™, Aidenbach, Germany) then fixed with 4% paraformaldehyde (Carl Roth GmbH, Karlsruhe, Germany) for 20 min at room temperature (RT). All of the following washing steps were conducted with prewarmed DPBS.

The immunofluorescence staining of *C. parvum* was conducted as previously described [35].

To stain the *G. duodenalis* trophozoites, cells were covered with the primary antibody mouse-anti-giardia (NativeAntigen, Kidlington, UK) in 1:200 dilution for 1 h. After washing, the secondary antibody goat-anti-mouse AlexaFluor™ 633 (Invitrogen, ThermoFisher Scientific, Waltham, USA) was applied on the cells for 45 min. Afterwards, the stain Hoechst 33342 (1:1000 ThermoFisher Scientific, Waltham, MA, USA) was added for 5 min, before the cells were washed again and finally covered with mounting medium (Fluoromount-G™ mounting medium, ThermoFisher Scientific, Waltham, USA). All slides were stored at 4 °C.

In case of co-infection, the secondary antibody goat-anti-mouse AlexaFluor™ 633 (Invitrogen, ThermoFisher Scientific, Waltham, USA) and the stain Sporo-Glo (Waterborne, New Orleans, USA) were applied to cells simultaneously.

The stained intra- and extracellular stages of *C. parvum* and *G. duodenalis* were analyzed with a Leica TCS SP8 DMi8 confocal laser scanning microscope (Leica Microsystems, Mannheim, Germany) equipped with the objective HC PL APO CS2 63×/1.30 GLYC and the software Leica Application Suite X (LAS-X 3.5.7.23225). Hoechst 33,342 was excited at 405 nm (PMT detection range 410–540 nm), Alexa Fluor 633 at 631 nm (HyD detection range 636–788 nm), and Cy3 at 561 nm (HyD detection range: 566–626 nm). Images along the *z*-axis with 31–66 layers (6.59–12.97 μm) were taken for purely illustrative purposes.

For additional processing of the acquired *z*-stacks, Huygens Professional software 20.10 (SVI, Hilversum, The Netherlands) was used for deconvolution, leading to minimized optical aberrations. To generate 3-D graphics of all *z*-stacks through surface rendering, Imaris software 10.10.1 (Oxford Instruments, Abingdon, UK) was utilized, and final intensity adjustments were completed with Photoshop CS 6 (Adobe, Dublin, Ireland).

### Statistics

To perform statistical analysis, the software GraphPad Prism 10.3.1 (GraphPad Software Inc., San Diego, USA) was used. The Shapiro–Wilk test and the Kolmogrov-Smirnov test were applied to determine normal distribution of all data.

Since the data were not normally distributed, the Kruskal–Wallis test or Friedmann test was applied. Both were followed by Dunn’s post hoc test.

*P* values < 0.05 were considered statistically significant. Significant levels are indicated as *p < 0.05, **p < 0.01, and ***p < 0.001.

## Results

### Infection of IPEC-J2 cells with *C. parvum* and CellTiter Blue cell viability assay

**Fig. 1:**
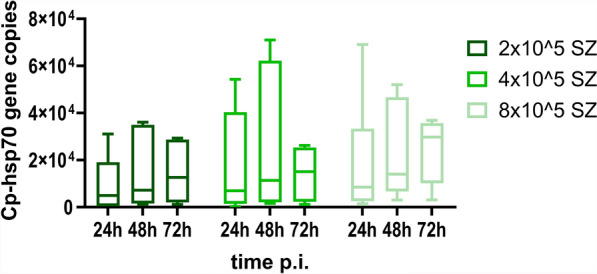
Mean number (± standard deviation) of Cp-hsp70 gene copies at three time points p.i. in IPEC-J2 cell cultures mono-infected with three doses of *Cryptosporidium parvum* sporozoites. SZ, *C. parvum* sporozoites/well; *n* = 6 except for 4 × 10^5^, 48 h p.i.; *n* = 5

**Fig. 2:**
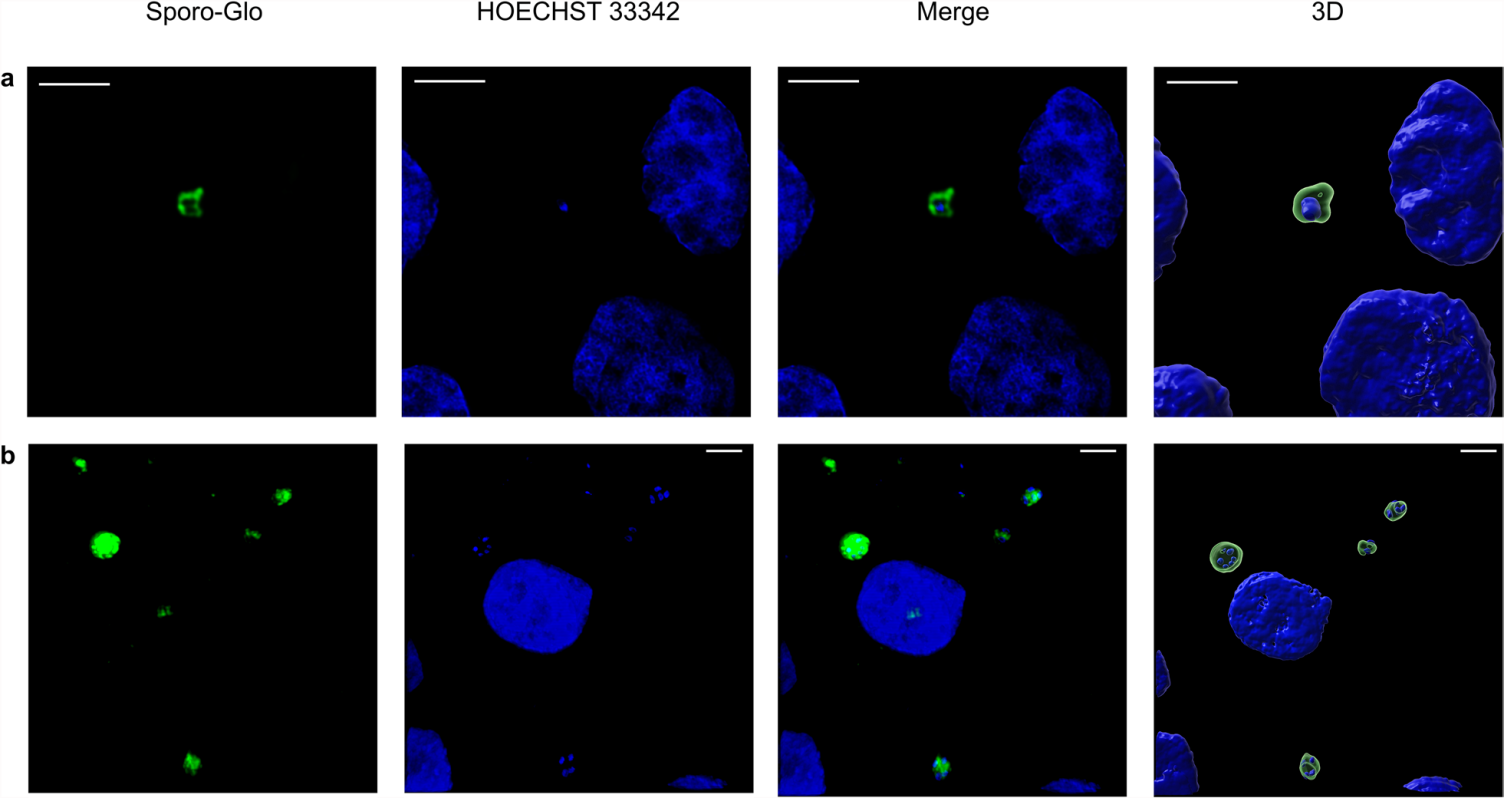
Maximum intensity projection images of *z*-stacks, showing a *C. parvum* trophozoite (**a**) and *C. parvum* meronts (**b**) using laser scanning microscopy on IPEC-J2 cell cultures infected with 8 × 10^5^ *C. parvum* sporozoites stained 48 h p.i. Nucleic acid of IPEC-J2 cells and parasite stages were stained with HOECHST 33342 (blue), the *C. parvum* trophozoites and meronts were stained with Sporo-Glo (green). Individual and merged channels are shown of each image, and Imaris was used to generate 3-D graphics by surface rendering solely for visualization purposes. Scale bar: 5 µm

**Fig. 3:**
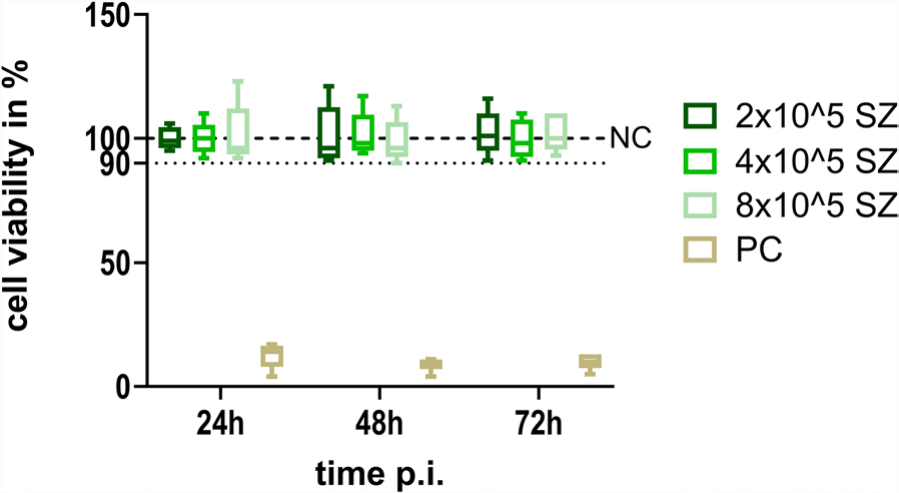
CellTiter Blue cell viability assay of IPEC-J2 cells infected with *C. parvum* sporozoites. NC, negative control, uninfected cultures; PC, positive control, cells cultured in PBS; SZ, *C. parvum* sporozoites/well; *n* = 5

Comparing three time points following mono-infection with various infection doses of *C. parvum* sporozoites, no significant variation of Cp-hsp70 gene copy numbers was found (Fig. 1). However, for infection doses of 2 × 10^5^ or 4 × 10^5^ sporozoites/well, gene copies tended to peak 48 h p.i.. Using immunofluorescence staining, it was observed that *C. parvum* sporozoites pursue their life cycle partially and develop into meronts (Fig. 2). Nevertheless, no significant impact on IPEC-J2 cell viability as compared with uninfected cultures was seen, irrespective of the amount of applied *C. parvum* sporozoites 24 h p.i., 48 h p.i., and 72 h p.i. (Fig. 3).

### MTT assay, infection of IPEC-J2 cells with *G. duodenalis* trophozoites, and CellTiter Blue cell viability assay

**Fig. 4:**
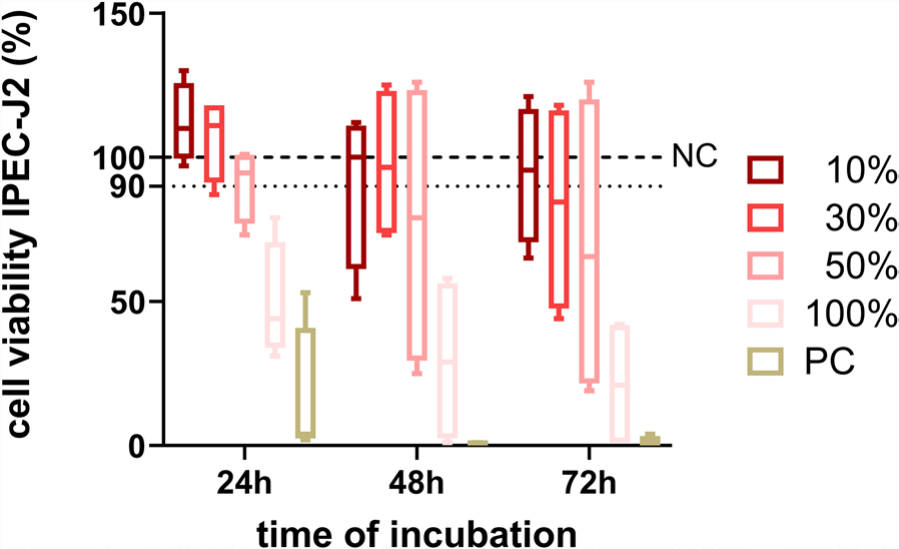
IPEC-J2 cell viability observed at three different time points during incubation with GM containing varying proportions of TYI-S-33** (**10, 30, 50 and 100%). PC, positive control, cells cultured in PBS; NC, uninfected negative control, cells incubated with GM only; *n* = 4

**Fig. 5:**
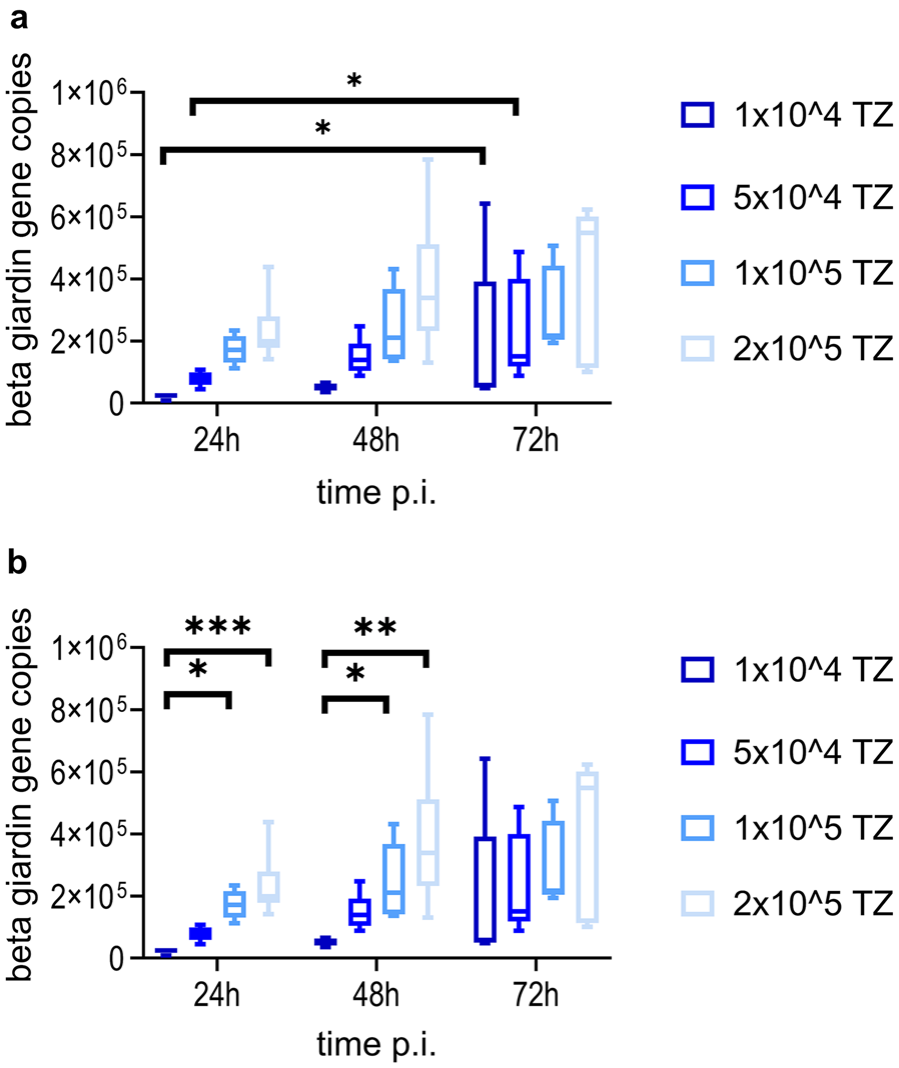
Mean number (± standard deviation) of β-giardin gene copies at three time points after infection with different amounts of *G. duodenalis* trophozoites. Comparing the development of infection doses over time (**a**) or the infection doses at the same measuring point (**b**). TZ, *G. duodenalis* trophozoites/well; *p < 0.05; **p < 0.01; ***p < 0.001; *n* = 5: 24 h p.i. with 1 × 10^4^ TZ or 1 × 10^5^ TZ, 72 h p.i. with 1 × 10^4^ TZ or 1 × 10^5^ TZ or 2 × 10^5^ TZ, all other samples: *n* = 6

**Fig. 6:**
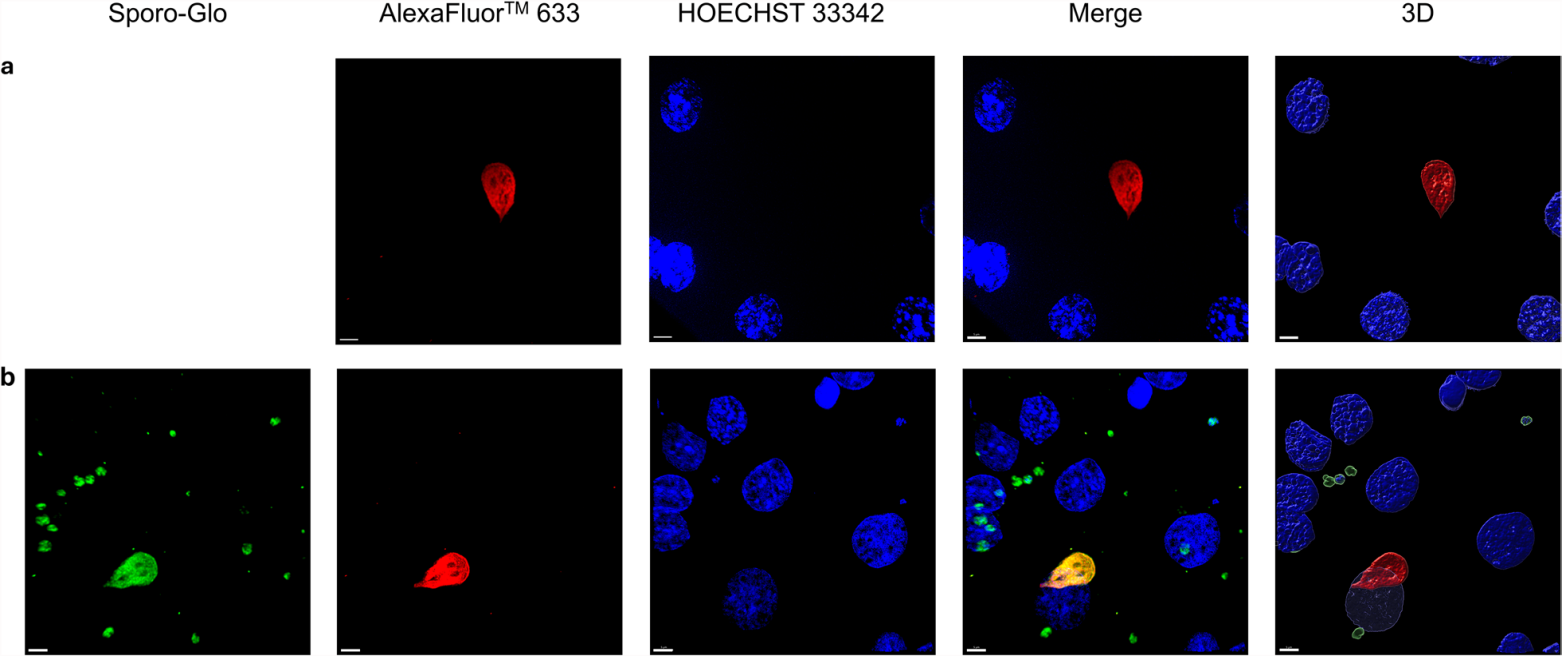
Maximum intensity projection images of *z*-stacks of mono-infection with *G. duodenalis* (**a**) and co-infection with *C. parvum* and *G. duodenalis* (**b**) obtained using laser scanning microscopy on IPEC-J2 cell cultures 48 h p.i. Nucleic acid of IPEC-J2 cells and parasite stages were stained with HOECHST 33342 (blue), the *C. parvum* stages were stained with Sporo-Glo (green), and *G. duodenalis* trophozoites were stained with the primary antibody mouse-anti-giardia and secondary antibody goat-anti-mouse AlexaFluor™ 633 (red). Owing to excitation crosstalk, the fluorophore Tropho-Glo gets also excited by the laser frequency aiming to excite Sporo-Glo, and thereby, emission of both fluorophores was detected in the same channel. Individual and merged channels are shown of each image, and Imaris was used to generate 3-D graphics by surface rendering solely for visualization purposes. Scale bar: 5 µm

**Fig. 7:**
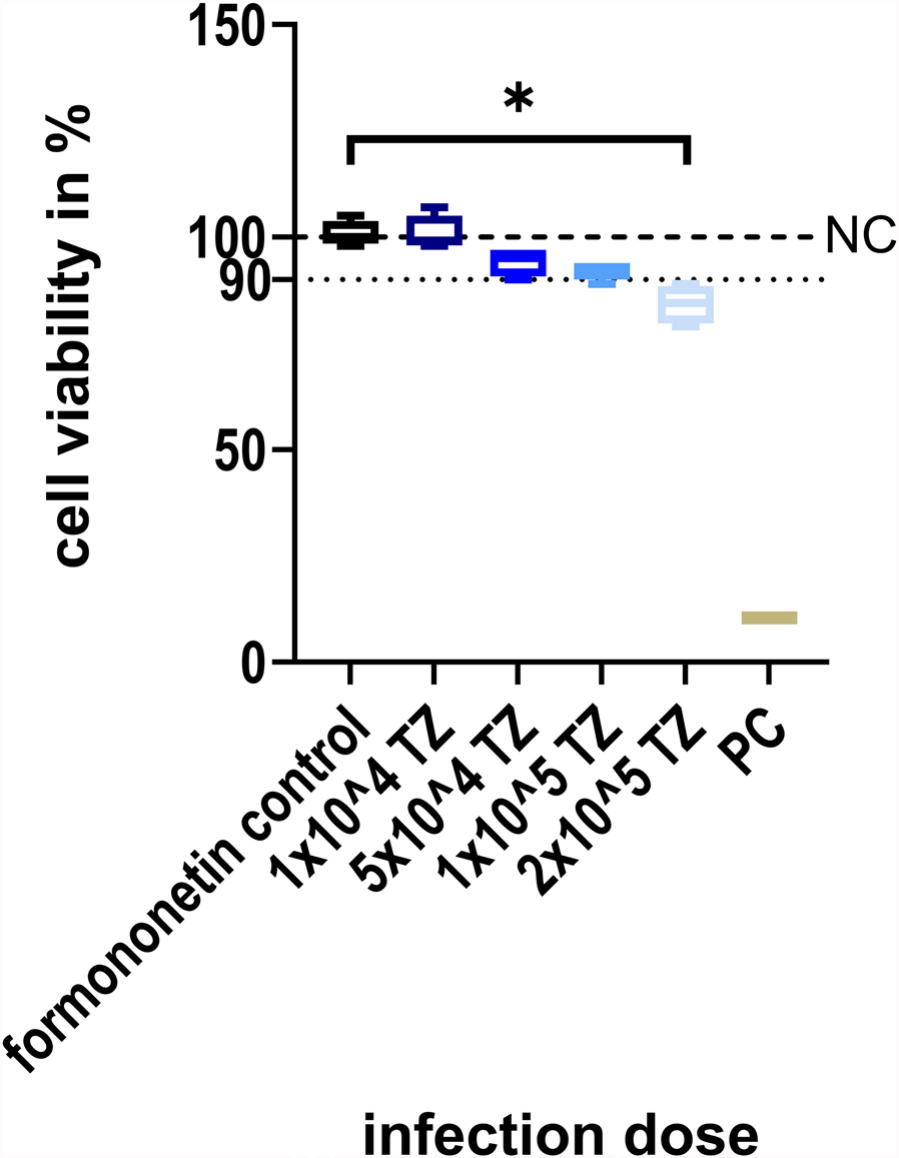
CellTiter Blue cell viability assay of IPEC-J2 cells infected with *G. duodenalis* trophozoites 72 h p.i. NC, negative control, uninfected and untreated cells; formononetin control, uninfected cells treated with formononetin only; PC, positive control, cells cultured with PBS; TZ, *G. duodenalis* trophozoites/well; *p < 0.05; *n* = 4

The use of GM containing 10% of the *Giardia* growth medium TYI-S-33 lead to a reduction of the median viability of IPEC-J2 cells, but not below a 90% level compared with NC over 72 h p.i. This stands in contrast to all higher amounts of TYI-S-33 tested (Fig. 4). Therefore, all infection experiments with *G. duodenalis* were performed using GM with 10% TYI-S-33. Light microscopic observation before collection of cells for DNA extraction showed that most trophozoites attached to IPEC-J2 cells, proving success of the infection (data not shown). *Giardia* infection with 1 × 10^4^ or 5 × 10^4^ trophozoites resulted in a significant increase of β-giardin gene copies 24 h p.i. and 72 h p.i. (Dunn’s multiple comparison, *Z* = 2.530, *P* = 0.0342 and *Z* = 2.309, *P* = 0.0418, respectively). The increase of β-giardin gene copies over 72 h p.i. with 1 × 10^5^ or 2 × 10^5^ trophozoites was not significant (Fig. 5a). A significant difference of β-giardin gene copies was observed 24 h p.i. when comparing the infection dose 1 × 10^4^ with those of 1 × 10^5^ (Dunn’s multiple comparison, *Z* = 3.237, *P* = 0.0072) or 2 × 10^5^ (Dunn’s multiple comparison, *Z* = 3.645, *P* = 0.0016). After 48 h p.i. β-giardin gene copies following infection with 1 × 10^4^ trophozoites differed significantly from those after application of 1 × 10^5^ (Dunn’s multiple comparison, *Z* = 3.058, *P* = 0.0134) as well as 2 × 10^5^ (Dunn’s multiple comparison, *Z* = 3.415, *P* = 0.0038). However, there were no significant differences between the infection groups at 72 h p.i. (Fig. 5b). Although many trophozoites detached during the immunofluorescence staining process, *G. duodenalis* was detected by laser scanning microscopy (Fig. 6a). *Giardia* trophozoites did not alter cell culture viability over 72 h p.i. at the lowest infection dose (1 × 10^4^), while the highest infection dose (2 × 10^5^) led to a reduction of the median viability below 90% compared with NC. Incubating the IPEC-J2 cells with formononetin for 30 min before performing the CellTiter Blue assay had no effect on cell viability (Fig. 7). Compared with the formononetin control, viability in cultures infected with 2 × 10^5^ *Giardia* trophozoites was significantly lower (Dunn’s multiple comparison, *Z* = 2.975, *P* = 0.0176) (Fig. 7).

### Co-culture model

**Fig. 8:**
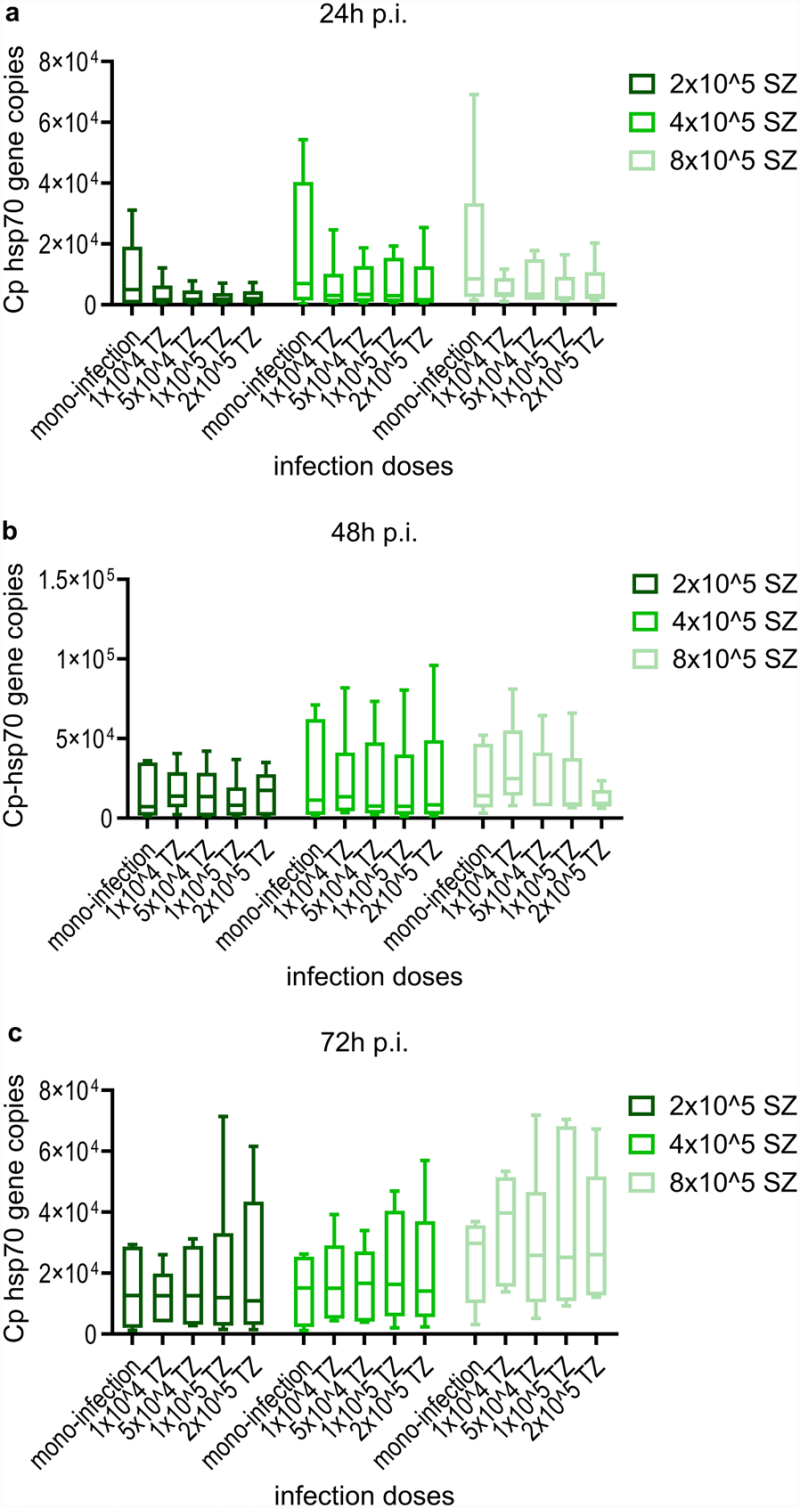
Quantification of Cp-hsp70 gene copies 24 h p.i.(**a**), 48 h p.i. (**b**), and 72 h p.i. (**c**), comparing mono-infections and co-infections using several infection doses; TZ, *G. duodenalis* trophozoites/well; SZ, *C. parvum* sporozoites/well; *n* = 5: 24 h p.i. with 4 × 10^4^ SZ and 5 × 10^4^ TZ or 1 × 10^5^ TZ, 48 h p.i. with 2 × 10^5^ SZ and 1 × 10^4^ TZ or 5 × 10^4^ TZ or 2 × 10^5^ TZ, 48 h p.i. with 8 × 10^5^ SZ and 5 × 10^4^ TZ, 72 h p.i. with 4 × 10^5^ SZ, 72 h p.i. with 8 × 10^5^ SZ and 2 × 10^5^ TZ; all other samples *n* = 6

**Fig. 9:**
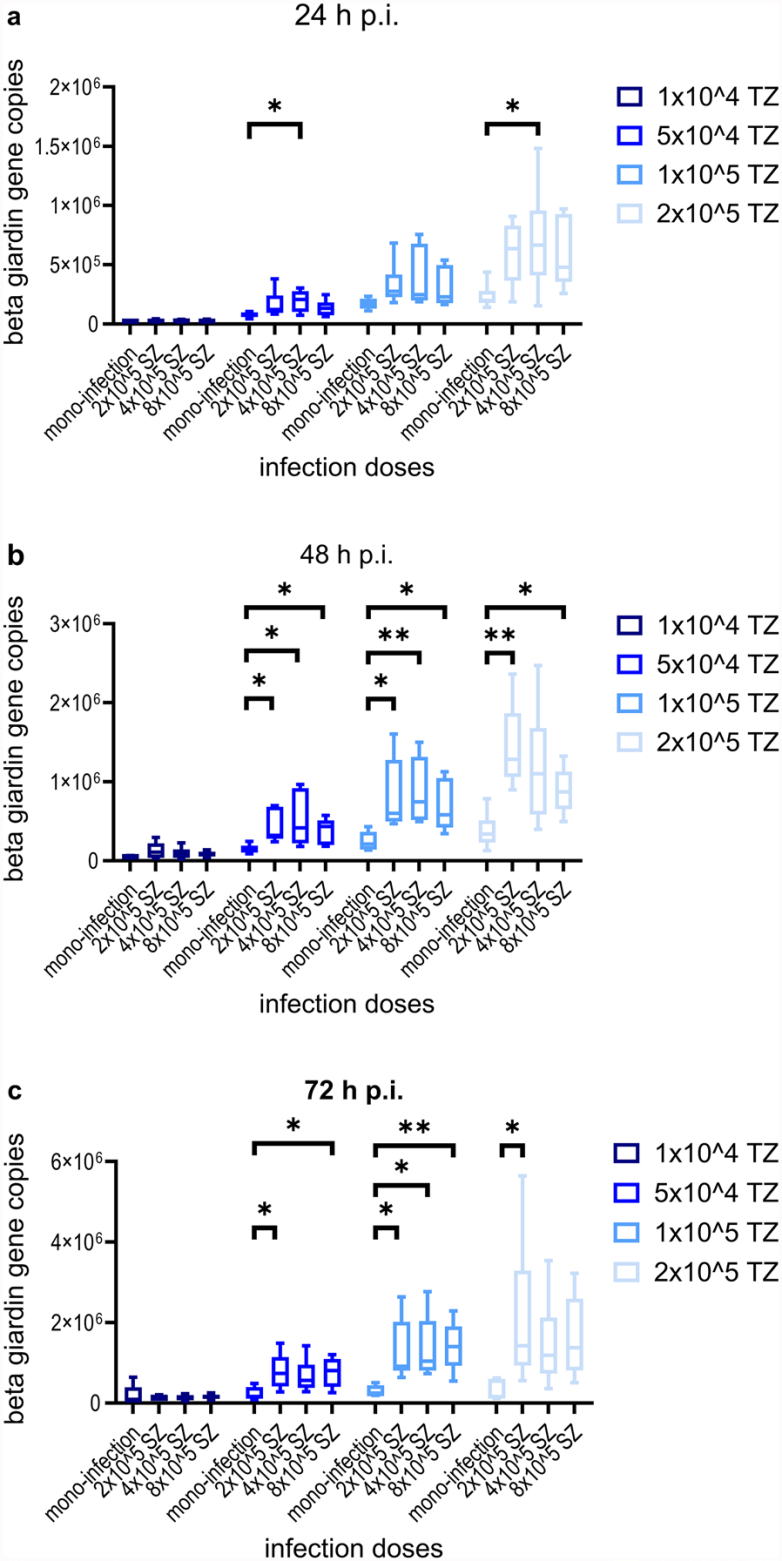
Quantification of β-giardin gene copies 24 h p.i. (**a**), 48 h p.i. (**b**), and 72 h p.i. (**c**), comparing mono-infections and co-infections using several infection doses; TZ, *G. duodenalis* trophozoites/well; SZ, *C. parvum* sporozoites/well; *p < 0.05; ** p < 0.01; *n* = 5: 24 h p.i. with 1 × 10^4^ TZ or 1 × 10^5^ TZ or 4 × 10^4^ SZ and 5 × 10^4^ TZ or 1 × 10^5^ TZ, 48 h p.i. with 1 × 10^4^ TZ or 2 × 10^5^ SZ and 1 × 10^4^ TZ or 5 × 10^4^ TZ or 2 × 10^5^ TZ or 8 × 10^5^ SZ and 5 × 10^4^ TZ, 72 h p.i. with 1 × 10^4^, 1 × 10^5^ or 2 × 10^5^ TZ or with 8 × 10^5^ SZ and 2 × 10^5^ TZ; all other samples *n* = 6

**Fig. 10:**
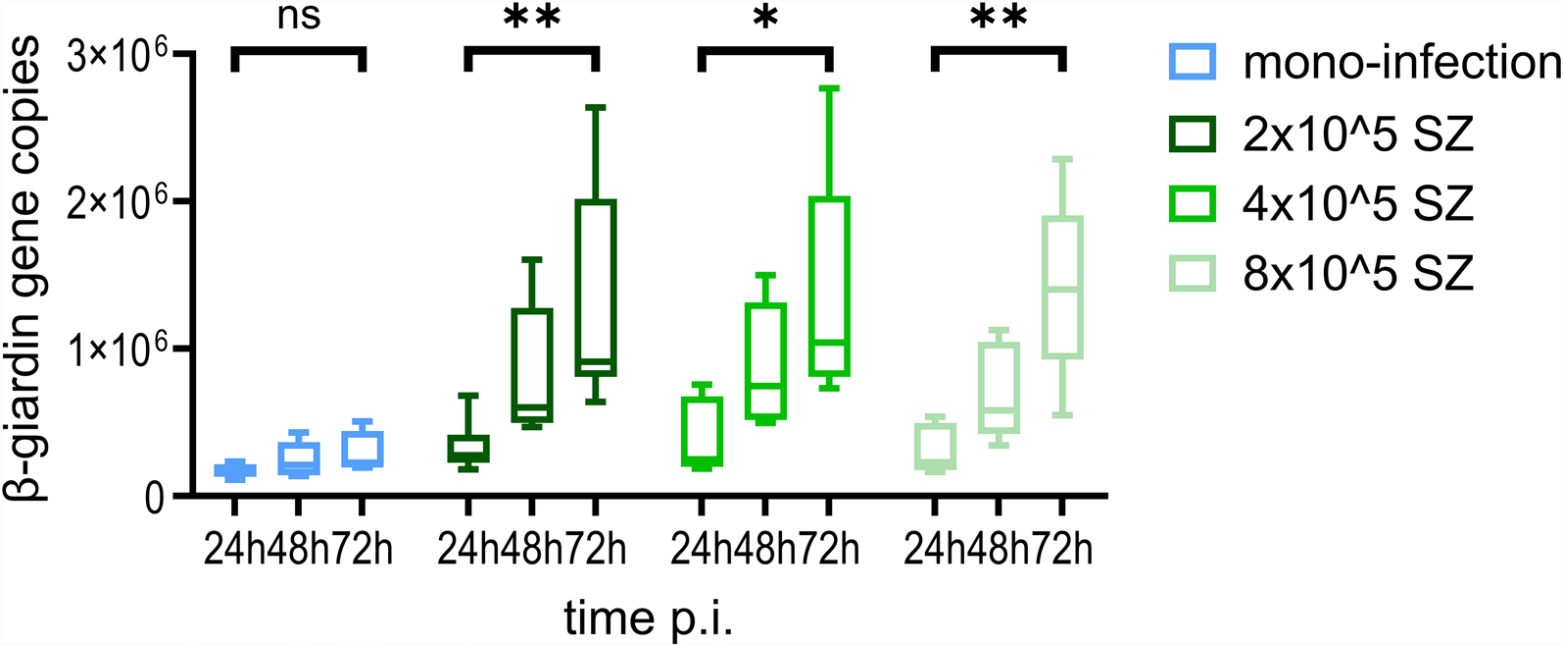
Quantification of β-giardin gene copies following infection of cell cultures with 1 × 10^5^ *G. duodenalis* trophozoites and various infection doses of *C. parvum* sporozoites at 24 h p.i., 48 h p.i., or 72 h p.i.; SZ, *C. parvum* sporozoites/well; *p < 0.05; **p < 0.01; *n* = 5: mono-infections at 24 h and 72 h p.i. and co-infection with 4 × 10^5^ SZ at 24 h; all other samples *n* = 6

Success of co-infection was visualized by immunofluorescence staining. No potential effect on the preferred localization of *G. duodenalis* trophozoites comparing mono- and co-infections could be determined owing to the detachment of many parasites during the staining process (Fig. 6b). Co-infection had no impact on *C. parvum* hsp70 gene copy numbers, and no significant differences between any co-infection groups and mono-infected cultures were seen over the observation period of 72 h p.i. (Fig. 8). In contrast, β-giardin gene copies and thus *Giardia* multiplication tended to increase and were even significantly increased in several co-infection groups as compared with *Giardia* mono-infected cultures (Fig. 9a–c). At 24 h p.i., cultures infected with 4 × 10^5^ *C. parvum* sporozoites and co-infected with either 5 × 10^4^ (Dunn’s multiple comparison, *Z* = 2.484, *P* = 0.039) or 2 × 10^5^ *G. duodenalis* trophozoites (Dunn’s multiple comparison, *Z* = 2.409, *P* = 0.048) displayed a significantly higher amount of β-giardin gene copies compared with mono-infected cultures with the same initial dose of *G. duodenalis* trophozoites. At 48 h p.i., significantly higher amounts of β-giardin gene copies than in mono-infected cultures were detected in cultures co-infected with 5 × 10^4^ (Dunn’s multiple comparison, *Z* = 2.646, *P* = 0.0244; *Z* = 2.724, *P* = 0.0193; Z = 2.469, *P* = 0.0407, respectively with increasing dose of *C. parvum* sporozoites) or 1 × 10^5^ (Dunn’s multiple comparison, *Z* = 2.899, *P* = 0.0112; *Z* = 3.184; *P* = 0.0044; *Z* = 2.409, *P* = 0.048, respectively with increasing dose of *C. parvum* sporozoites) *G. duodenalis* trophozoites, irrespective of the infection dose of *C. parvum*. Cells co-infected with 2 × 10^5^ trophozoites of *G. duodenalis* and 2 × 10^5^ (Dunn’s multiple comparison, *Z* = 3.16, *P* = 0.0047) or 4 × 10^5^ (Dunn’s multiple comparison, *Z* = 2.667, *P* = 0.0229) *C. parvum* sporozoites differed significantly from mono-infected cultures in terms of higher β-giardin gene copy numbers. At 72 h p.i., the amount of β-giardin gene copies was significantly increased in cultures simultaneously infected with either 5 × 10^4^ (Dunn’s multiple comparison, *Z* = 2.531, *P* = 0.0341) or 1 × 10^5^ (Dunn’s multiple comparison, *Z* = 2.962, *P* = 0.0092) *G. duodenalis* trophozoites and 8 × 10^5^ *C. parvum* sporozoites compared with mono-infections. Additionally, significantly higher gene copy numbers were detected in cells co-infected with 2 × 10^5^ *C. parvum* sporozoites and 5 × 10^4^ (Dunn’s multiple comparison, *Z* = 2.613, *P* = 0.0269), 1 × 10^5^ (Dunn’s multiple comparison, *Z* = 2.638, *P* = 0.025) or 2 x 10^5^ (Dunn's multiple comparison, Z = 2.518, *P* = 0.0354) *G. duodenalis* trophozoites or in cultures co-infected with 4 × 10^5^ *C. parvum* sporozoites and 1 × 10^5^ (Dunn’s multiple comparison, *Z* = 2.8, *P* = 0.0153) *Giardia* trophozoites. However, no significant effect was seen in comparison with cultures infected with 1 × 10^4^ *G. duodenalis* trophozoites at any measuring point. Altogether, irrespective of the applied *C. parvum* dose, co-infected cultures with 1 × 10^5^ *Giardia* trophozoites displayed a significant increase of β-giardin gene copies during the observation period until 72 h p.i. in contrast to mono-infected cultures (Dunn’s multiple comparison, *Z* = 3.175, *P* = 0.0045; *Z* = 2.887, *P* = 0.0117; *Z* = 3.175, *P* = 0.0045, respectively with increasing *C. parvum* dose) (Fig. 10).

## Discussion

Several recent studies investigated *Giardia* and *Cryptosporidium* infections in cattle [36–39]. While *C. parvum* infection is undoubtedly a cause for diarrhea in calves [40], the clinical effect of *Giardia* sp. infections in livestock remains unclear. Gao et al. could not show a correlation between *Giardia* infection and diarrhea in calves [40]. Clinical disease due to *Giardia* infection occurs in humans; however, it was shown that over 50% of *G. duodenalis* infections in humans do not lead to clinical symptoms at all [41–43]. On the other hand, in calves, a significant association between *Giardia* and diarrhea and a negative influence on the weight gain in even asymptomatic calves was reported [36, 44]. Co-infections with both parasites have been reported [9, 39, 45–47], and Mateusa et al. demonstrated a notable correlation between *Cryptosporidium* spp. and *G. duodenalis* co-infection in dogs, concluding that the presence of one of these parasites elevates the chance of infection by the other [48]. Still there is little knowledge about co-infection’s impact on the course of infection or on the severity of clinical symptoms. To investigate possible mutual effects on the course of co-infections by *C. parvum* and *G. duodenalis*, a cell culture model with IPEC-J2 cells was established and the course of cell culture infection monitored over 72 h. For co-infection, *C. parvum* sporozoites were applied to confluent host-cell monolayers initially, and 3.5 h later, *G. duodenalis* trophozoites were added. This protocol is in line with previous studies showing that *C. parvum* is frequently detected earlier than *Giardia* in the field [45, 49–51]. Successful single infections of IPEC-J2 cells with *C. parvum* have been described previously [24, 25]. On the basis of our former work [24], an optimized infection protocol according to Berberich was applied [35]. Infection by application of 2 × 10^5^ or 4 × 10^5^ *C. parvum* sporozoites did induce a not significant peak of infection 48 h p.i., which is in line with Delling et al. and Ferguson et al. [24, 25]. Holzhausen et al. detected a great variability in the cytopathogenicity depending on infection doses and isolates of *C. parvum* in HTC-8 cells [31]. The *C. parvum* isolate used in this study contain the zoonotic subtype family IIa, which predominantly infects cattle [3]. Especially the subtype IIaA15G2R1 is a commonly identified subtype in European livestock and human cases and known for its high transmissibility [3]. To asses potential impairment of IPEC-J2 cell viability due to *C. parvum* sporozoites in our current model, cell viability assays were conducted considering all three infection doses selected. No significant negative impact on the host cell viability could be observed at 24 h p.i., 48 h p.i., and 72 h p.i. This in line with a previous study proving 4–8 × 10^5^ *C. parvum* sporozoites to be adequate for infection studies in 24-well plates using the cell lines HCT-8 and COLO-680N [35]. Additionally, it was shown that even smaller amounts of *C. parvum* sporozoites can cause a traceable infection in IPEC-J2 cells. Immunofluorescence staining proved suitable to track the infection [35], confirming successful infection by *C. parvum*. Bénéré et al. and Fisher et al. demonstrated that axenic cultivation of *Giardia* trophozoites requires microaerobic conditions [52, 53]. Nevertheless, in vitro models for culturing of *G. duodenalis* trophozoites with intestinal cell lines require aerobic conditions and, in contrary to axenic culture, *G. duodenalis* trophozoite cultivation is possible under aerobic conditions when co-cultured with intestinal cells [53–55]. Previous studies on cell culture of these parasites were mostly performed with transformed permanent cell lines [56–59]. In the current study it was shown that nontransformed porcine IPEC-J2 cells are well suited to study both *C. parvum* and *G. duodenalis* in vitro. Previous studies have shown that the cell line IPEC-J2 offers a biologically relevant model for studies of zoonotic enteric infections mimicking the in vivo situation of the small intestinal physiology [21]. Monolayers of this cell line have already been used for investigating interactions with several parasites, including *C. parvum* [22–25]. In previous studies *Giardia* infected cell cultures were maintained for 24 h and even longer [53, 60, 61]. Fisher et al. stated that *Giardia* proliferation is not supported when cultured with CaCo-2 cells grown in 100% DMEM, while other media combinations strongly affected the course of proliferation [53]. In our study, TYI-S-33 was used to promote *Giardia* growth, and the effect of different medium combinations on IPEC-J2 cells was tested. A mixture of 90% GM and 10% TYI-S-33 appeared suitable for culture of host cells and parasites in our hands, resulting in a significant proliferation of *Giardia* trophozoites for 72 h p.i. Proliferation of *Giardia* trophozoites depended on the particular infection dose used. Interestingly, the lowest infection dose (1 × 10^4^) led to a significant increase of gene copies from 24 h p.i. to 72 h p.i., whereas the highest infection dose (2 × 10^5^) did not result in a significant enhancement of the amount of gene copies over the observation period. This indicates a limitation of proliferation depending on the initial amount of trophozoites used and may be linked to limitation of nutrients provided in the medium, as demonstrated by Fisher et al. [53]. These authors stated that certain nutrients contained in TYI-S-33 are essential for trophozoites to grow and may not be provided in medium TYI-S-33 in a sufficient amount to allow proliferation of large numbers of trophozoites. Another explanation could be a saturable amount of binding sites and/or host cell surface receptors for attachment of trophozoites. Müller et al. conducted an attachment assay with *Giardia* on Caco2 cells and observed 20–40% less attached trophozoites within 24 h p.i. when 10^6^ trophozoites were applied instead of 10^5^ trophozoites per well [61], supporting our finding of dose-dependent trophozoite proliferation. To examine the effect of a *Giardia* infection on IPEC-J2 cell viability and to assess potential differences between the various infection doses, a CellTiter Blue viability assay was performed. To remove the trophozoites from the cultures, formononetin was used, which was demonstrated not to affect host cell viability in the current study as well as in previous publications [53, 62]. However, a reduction of host cell viability when a high amount of *Giardia* trophozoites was applied obviously appeared. The results concerning the reduced cell viability are in line with former studies showing that *Giardia* trophozoites cause epithelial alterations that may lead to a disruption of the intestinal barrier [63, 64]. One may speculate that higher quantities of trophozoites lead to more cell alteration and thus may be related to more severe pathophysiological effects and clinical symptoms under in-vivo conditions. Co-infections by several pathogens are frequently found in the field and may modify the infection course, as was shown for cryptosporidiosis. Bednarska et al. described an increased number of shed oocysts during co-infection with the nematode *Heligmosomoides bakeri* in mice [65]. Piglets infected with *C. parvum* and rotavirus showed more severe clinical symptoms, elevated fecal oocyst counts, and greater intestinal damage than animals infected with *C. parvum* alone [66, 67]. Studies in cattle delivered similar observations showing that co-infections of *Cryptosporidium* sp. and rotavirus or coronavirus more likely resulted in diarrhea as compared with mono-infections with either of these pathogens [68, 69]. Bajer et al. reported a higher prevalence of *C. parvum* and *Giardia* spp. infections in wild mice when a helminth infection occurred at the same time [70]. Lambs passed more soft feces if tested positive for both *Cryptosporidium* and *Giardia* [71]. In contrast, co-infection with *Trichinella* led to a reduction of the number of *Giardia* cysts shed in the feces of mice, possibly owing to a decreased villus-to-crypt ratio leading to a reduced surface area for trophozoite attachment and causing trophozoites to be more susceptible to intestinal peristalsis, whereas other factors such as competition for essential nutrients, secretion of toxic products, or an increased immune response cannot be excluded [72]. Taha et al. examined the course of co-infection by *C. parvum* and *Eimeria acervulina* in a chicken macrophage cell line and showed significantly reduced multiplication of *E. acervulina* in co-infected cell cultures, while multiplication of *C. parvum* was not significantly affected [18]. A similar lack of significant effects on the quantity of Cp-hsp70 gene copies between mono-infections and co-infections of *C. parvum* and *Giardia* was observed in the current study. Thus, both models showed a change in multiplication of the respective protozoa during co-infection with *C. parvum*, while *C. parvum* multiplication appears to remain unaffected by the other pathogen. However, it has to be considered that *E. acervulina* is an intracellular parasite, similar to *C. parvum*, whereas *G. duodenalis* multiplies extracellularly. Therefore, interactions with *C. parvum*, although not well defined so far, do most likely differ, and comparison of results obtained from these models has to be done with care, especially concerning possible competitive factors for essential nutrients during infection. While *C. parvum* is located within a parasitophorous vacuole and relies heavily on glucose passing through the host cell’s feeder organelle as its main energy source [73], the extracellularly attached *G. duodenalis* trophozoites do not depend on glucose as a metabolic substrate, but can also use arginine or aspartate for nutritious purposes [74, 75]. Former studies observed an influence of *C. parvum* infection on the intracellular glucose levels of host cells [24, 76]. One may speculate that the enhanced proliferation of *G. duodenalis* trophozoites during co-infection was associated to the elevated glucose consumption by *Cryptosporidium*-infected host cells, resulting in enhanced use of l-arginine by *Giardia* as a primary energy source. Consumption of l-arginine results in decreased nitric oxide (NO) production by the epithelial cells, which was shown to be important in *Giardia* eradication in mice [77–80]. Interestingly, it was shown previously that chronic *G. duodenalis* infections led to reduced sodium-coupled d-glucose absorption in the duodenum of human patients owing to a reduction in the villous surface area [81]. Since trophozoite multiplication was increased in the presence of *C. parvum* and an increase in trophozoite numbers reduced the viability of host cells, it might be speculated that such effects could also aggravate intestinal lesions in natural hosts. However, in this study, infections with both parasites were maintained in vitro for only 72 h, and therefore, conclusions on effects during chronic co-infection in vivo should be drawn reluctantly. Studies on potential influence on, e.g., host cell metabolism or production of NO remain to be performed. To investigate pathomechanisms and effects of *Cryptosporidium*–*Giardia* co-infection in more detail and under conditions more similar to the in vivo situation, further investigations using a 3-D model may be reasonable. Holthaus et al. investigated co-infection by *G. duodenalis* and *Toxoplasma gondii* in murine organoid-derived cultures obtained from duodenal crypt samples [64]. While *G. duodenalis* showed a great influence on, e.g., transepithelial electrical resistance (TEER) and integrity of the host cells, *T. gondii* did not impact either the host cells or the other pathogen in case of co-infection. Since alterations of the barrier function did not differ between co-infection and *Giardia* mono-infection, one may speculate that *T. gondii* does not enhance *Giardia* proliferation, although no quantification of parasites was conducted in this cited study. Successful infection of organoid models with *C. parvum* has also been described previously [19], enabling the parasite to complete its lifecycle and to prolong the cultivation period. Therefore, investigations of parasites’ proliferation under those conditions might help to support our findings and give deeper insight in parasite–host interactions.

## Conclusions

Mono- and co-infected cultures were maintained over a period of 72 h under the conditions described above. Although co-infections by *Giardia* spp. and *Cryptosporidium* spp. have been reported in the field previously, this is the first in vitro co-infection study in intestinal cells, to the best of our knowledge. Proliferation of *G. duodenalis* trophozoites attached to IPEC-J2 cells and formation of *C. parvum* meronts were detected during simultaneous infection. Co-infections with both protozoan parasites led to significantly higher replication of *G. duodenalis*, whereas effects on *C. parvum* were not observed. The pathomechanism behind these results and conclusions on whether these two parasites interact similarly under in vivo conditions remain to be demonstrated.

## Data Availability

Data are provided within the manuscript.

## Abbreviations

BC: Blank control
DPBS: Dulbecco's phosphate-buffered saline w: Ca/Mg
DMEM: Dulbecco's modified Eagle's medium
FCS: Fetal calf serum
F-12: Ham’s F-12 nutrient mixture W/GlutaMAX™-I
GM: Growth medium
IMDM: Iscove’s modified Dulbecco’s medium
NC: Negative control
OD: Optical density
PC: Positive control
PBS: Phosphate-buffered saline
p.i.: Post infection
qPCR: Quantitative real-time polymerase chain reaction
3-D: Three dimensional
